# Transportan 10 Induces Perturbation and Pores Formation in Giant Plasma Membrane Vesicles Derived from Cancer Liver Cells

**DOI:** 10.3390/biom13030492

**Published:** 2023-03-07

**Authors:** Sara Anselmo, Giuseppe Sancataldo, Concetta Baiamonte, Giuseppe Pizzolanti, Valeria Vetri

**Affiliations:** 1 Dipartimento di Fisica e Chimica-Emilio Segré, Università degli Studi di Palermo, 90128 Palermo, Italy; 2 Dipartimento di Scienze e Tecnologie Biologiche Chimiche e Farmaceutiche, Università degli Studi di Palermo, 90128 Palermo, Italy; 3 AteN Center-Advanced Technologies Network Center, Università degli Studi di Palermo, 90128 Palermo, Italy; 4 Dipartimento di Promozione della Salute, Materno-Infantile, di Medicina Interna e Specialistica di Eccellenza “G. D’Alessandro”, Università degli Studi di Palermo, 90127 Palermo, Italy

**Keywords:** protein–membrane interactions, cell-penetrating peptides, Transportan 10, giant plasma membrane vesicles, phasor approach, Nile Red, di-4-ANEPPDHQ, membrane hydration

## Abstract

Continuous progress has been made in the development of new molecules for therapeutic purposes. This is driven by the need to address several challenges such as molecular instability and biocompatibility, difficulties in crossing the plasma membrane, and the development of host resistance. In this context, cell-penetrating peptides (CPPs) constitute a promising tool for the development of new therapies due to their intrinsic ability to deliver therapeutic molecules to cells and tissues. These short peptides have gained increasing attention for applications in drug delivery as well as for their antimicrobial and anticancer activity but the general rules regulating the events involved in cellular uptake and in the following processes are still unclear. Here, we use fluorescence microscopy methods to analyze the interactions between the multifunctional peptide Transportan 10 (TP10) and the giant plasma membrane vesicles (GPMVs) derived from cancer cells. This aims to highlight the molecular mechanisms underlying functional interactions which bring its translocation across the membrane or cytotoxic mechanisms leading to membrane collapse and disruption. The Fluorescence Lifetime Imaging Microscopy (FLIM) method coupled with the phasor approach analysis proved to be the winning choice for following highly dynamic spatially heterogeneous events in real-time and highlighting aspects of such complex phenomena. Thanks to the presented approach, we were able to identify and monitor TP10 translocation into the lumen, internalization, and membrane-induced modifications depending on the peptide concentration regime.

## 1. Introduction

In the last years, natural and synthetic membrane-active peptides have gained increasing interest for their biological role in host defense [1,2,3,4]. These peptides carry out their biological activity by interacting with membranes according to different molecular pathways. Peptides may induce membrane structural modifications [5,6], may be adsorbed at their interface with minimal effect [7], and may deliver molecules across biological membrane barriers [8,9,10]. Among them, the family of short (fewer than 30 amino acids) cell-penetrating peptides (CPPs) is arousing great interest due to their biocompatibility and potentially controllable physico-chemical properties. CCPs have shown the ability to efficiently cross cell membranes without inducing alterations also facilitating the translocation of drugs, bound through covalent or electrostatic interactions [10,11,12,13,14,15]. Thanks to this peculiar behavior, these membrane-active peptides have shown multiple potential applications in the diagnosis and therapy of diseases which include cancer, inflammation, and central nervous system disorders [16]. Furthermore, some recent work has highlighted a direct antibacterial [4,17] and/or anticancer activity [10,18] of some peptides belonging CPPs family. These results are of great interest in the field even if the mechanisms of action are not completely clear yet. Different types of translocation processes through the membrane were suggested and described [19]. In general, the internalization of these peptides and concurrent membrane structural modifications have been shown to be dependent on several interdependent physicochemical parameters such as temperature, specific properties of the membrane [20], peptides sequence, and concentration [7,21,22]. Moreover, the understanding of the fundamental biophysical determinants of the interactions between a peptide and membrane is crucial to improve the knowledge of bio-membrane functions such as membrane transport, fusion, and signaling processes and to shed light on potential applications of membrane-active peptides. This knowledge may help in devising general rules helping the rational design of new membrane-targeting therapeutic agents that could fight the evolution of drug resistance [1]. Electrostatic interactions between charged amino acids of the polypeptide chain and head groups of the lipid bilayer, in cooperation with hydrophobic interactions which imply dehydration of apolar aminoacidic residues, are believed to underlie the action of these peptides. However, the high level of complexity of cell membranes makes the detailed analysis of the molecular mechanisms, involved in the peptide–membrane interactions, a real challenge.

In this work, we analyze the interaction between Transportan 10 (TP10), a widely studied synthetic CPP, and giant plasma membrane vesicles (GPMVs) directly derived from cancer liver cells. Fluorescence lifetime imaging measurements are used to unravel occurring events. TP10 is an amphiphilic, α-helix, cationic CPP, constituted by 21 amino acids. The presence of four lysines, which impart it positive charge, together with the absence of negatively charged amino acids and the presence of the N-terminus, results in a global formal charge of +5 at neutral pH [23]. It is known that TP10 is able to cross plasma membranes also by translocating molecular cargoes [24,25]. It was demonstrated that TP10 presents high pharmaceutical potential in vitro and in vivo as a carrier for proteins (streptavidin, avidin) [26] but also nucleic acids, opening an intriguing perspective for gene delivery applications [15,27,28]. In addition, it was found that the ability of this peptide to cross cell membranes can result in toxic effects as a consequence of membrane perturbation at high peptide concentrations [4]. To date, various mechanisms of penetration and membrane modification induced by TP10 and by other similar peptides able to act on membranes have been proposed, which also involve conformational modifications of the peptide [29]. The penetration is believed to involve both endocytosis and direct translocation [13,19], while transient modifications of the membrane were found to be induced by competing mechanisms, often acting in parallel, which include carpeting, the formation of sinking rafts or toroidal pores [23,30]. It was reported that, in the extracellular environment, TP10 assumes an α-helical structure due to interactions with charged and hydrophobic membranes [31]. Interacting with membranes, the helical structure of TP10 is initially oriented parallel to the membrane surface, while the hydrophobic residues of the bounded peptide displace the polar head groups, creating a breach in the hydrophobic region and inducing a change in the curvature strain in the membrane [6,31,32]. The introduction of strain and thinning further destabilizes the membrane surface integrity, making it more vulnerable to subsequent peptide interactions. When a certain peptide-to-lipid ratio threshold is overcome, peptides may orient perpendicular to the membrane, helices begin to self-associate, such that their polar residues are no longer exposed to the membrane hydrocarbon chains, and pores start to form [6]. The multiple mechanisms leading TP10 to exploit its activity and their complex dependence on the peptide’s physicochemical properties, cell peculiarities, and environmental conditions are the subject of a large number of studies, but a clear picture has not been achieved yet.

Recently, we have shown a concentration-dependent effect of TP10 on model membranes which results in the absorption or insertion of this peptide in the membrane bilayer [7] with a consequential reduction of the fluidity of the membrane and its dehydration. This can induce membrane weakness and instability which may explain the altered behavior of cells and microbials observed in the presence of this peptide. Moreover, and importantly for functional implications, we have also shown that TP10 does not interact with membranes enriched with cholesterol [7].

In this work, the effect of TP10 was studied on more complex model membranes, i.e., GPMVs directly derived by HepG2 cells, a liver cancer cell line. This choice also derived from the discovery of the cytotoxic and selective effects of TP10 against cancer cells [26,33] (in particular, erytroblastic leukemia and breast cancer). The selectivity toward cancer cells was attributed to the higher concentration of negatively charged phospholipids, such as phosphatidylserine (PS) and phosphatidylethanolamine (PE), which are exposed in the outer membrane together with the overexpression of anionic glycoprotein and altered fluidity [34].

The use of GPMVs presents the advantage of having a stable model system with similar composition, in terms of protein and lipids, to intact cell plasma membranes and with cytoplasm inside [35,36]. These model membranes are characterized by a defined inner cavity volume and long-term stability, so they constitute an excellent model in biophysical studies for the analysis of molecular interactions, which are difficult to measure in live cells due to the complex reactions related to a functional activity that may hinder repeatability. These structures provide an invaluable tool with reduced artifact possibilities that can be caused by concomitant endo- and exo-cytosis processes and disturbances from intracellular dynamics. Using Fluorescence Lifetime Imaging Microscopy (FLIM) and monitoring the lifetime changes in suitable fluorescence molecular reporters such as Nile Red (NR) and di-4-ANEPPDHQ, we were able to evaluate the interaction between TP10 and membranes, analyzing both the fate of TP10 and the changes in morphology/integrity, hydrophobicity and fluidity of the membranes. NR was selected since its spectral properties (absorption and fluorescence spectra, quantum yields, and lifetime) strongly depend on the polarity of the environment [37,38] and they can be suitably complemented with membrane fluidity data resulting from di-4-ANEPPDHQ. The latter is in fact a dye commonly used to monitor changes in the physical order of membranes, ref. [39,40,41] providing a tool to extrapolate molecular details on the occurring events.

## 2. Material and Methods

### 2.1. Materials

The HepG2 cell line was purchased from the American Type Culture Collection. Fetal bovine serum was purchased by EUROCLONE (ECS0180DH), while L-glutamine (25030081) and penicillin-streptomycin (15140122) were purchased by GIBCO. DMEM Medium (D6046), Sodium chloride (NaCl) (S9888), Calcium chloride (CaCl_2_) (C1016), HEPES (H3375), Hydrochloric acid (HCl) (1.01514), Sodium hydroxide (NaOH) (S8045), Paraformaldehyde (PFA) (16005), Dithiothreitol (DTT) (D9779), and Nile Red (19123) were purchased from Sigma-Aldrich (Sofia, Bulgaria). Transportan 10 (TP10) and TP10 labeled with carboxyfluorescein (CF) at the N-terminus (CF-TP10) were purchased from the CRIBI-Peptide Facility, University of Padova. di-4-ANEPPDHQ (D36802) was purchased from Thermo Fisher Scientific (Waltham, MA USA).

### 2.2. Giant Plasma Membrane Vesicles (GPMVs) Preparation and Staining

HepG2 cells were cultured in Dulbecco’s Modified Eagle Medium supplemented with 10% fetal bovine serum, 2 mM L-glutamine, and 100 units/mL of penicillin–streptomycin at 37 °C in a humidified 5% CO_2_ and were passaged once a week at 1:2 split ratio. To obtain GPMVs, cells were used at about 70% confluence. Briefly, HepG2 cells were treated, according to the protocol [35], with GPMV forming buffer (10 mM HEPES, 150 mM NaCl, 2 mM CaCl_2_, pH 7.4) containing vesiculation agents (25 mM PFA (4% (*w*/*v*)/2 mM DTT (1 M)) and were imaged using microscopy (Axio Observer.D1, Zeiss, Oberkochen, Germany) and incubated for 1 h at 37 °C. After vesicle formation, GPMVs were separated from adherent cells by transferring the supernatant into Eppendorf tubes using pipetting. Although most cells remain attached to the flask, cellular debris in the supernatant was separated from GPMVs using differential centrifugation (100× *g* for 10 min). In line with the literature, for fluorescence microscopy experiments, the GPMV suspensions were added to Nile Red (NR) and di-4-ANEPPDHQ in a final concentration of 4 μM and allowed 1.5 h for the incorporation of the dyes prior to any experiment.

### 2.3. Fluorescence Microscopy

All confocal fluorescence microscopy measurements were performed using a Leica TSC SP5 confocal laser scanning microscope with a 63×/1.4 oil objective (Leica Microsystems, Wetzlar, Germany) and with a scanning frequency of 400 Hz.

#### 2.3.1. Co-Localization Experiments

Images of GPMVs at 1024 × 1024 pixels resolution, unstained or stained with Nile Red (NR), after adding CF-TP10 (3 μM and 10 μM) were collected. The fluorescence signal of CF-TP10 was detected in the range of 500–650 nm (λ_exc_ = 470 nm), while the NR fluorescence signal was detected in the range of 580–700 nm (λ_exc_ = 540 nm).

#### 2.3.2. Fluorescence Lifetime Imaging Microscopy (FLIM)

Before and after peptide addition, 256 × 256 pixels FLIM images were acquired in the time domain using the Leica TCS SP5 microscope coupled with a PicoHarp 300 TCSPC Module (PicoQuant, Berlin, Germany). The fluorescence signal of CF and di-4-ANEPPDHQ was collected in the range of 500–650 nm using λ_exc_ = 470 nm. For Nile Red, λ_exc_ = 540 nm and its fluorescence signal were collected in the range of 580–700 nm. The pulsed and tunable White Light Laser (Leica Microsystem) source was used in these experiments.

### 2.4. FLIM Phasor Analysis and Interpretation

The phasor approach was used to analyze FLIM data [42]. Phasor analysis is a fit-free Fourier domain technique that converts the fluorescence decay measured in each pixel of the image to a single point called “phasor” in a calibrated polar plot (phasor plot): a semicircle with radius ½ drawn from point (0, 0), corresponding to τ = ∞, to point (1, 0), corresponding to τ = 0, which is commonly referred as a “universal circle”. In this representation, the single exponential decays fall along the circumference while complex decays generate phasors (or phasor distributions) within the circle. A one-to-one correspondence exists between pixels in the FLIM image and pixels in the phasor plot so that, by selecting pixels in the phasor plot using colored cursors, the corresponding pixels in the measurement will be colored accordingly generating the so-called “lifetime maps” [42,43]. Importantly, since the phasors follow the rules of vector algebra, it is possible to geometrically resolve the fractions in the fluorescence decay components with the lever rule of vector additions. Indeed, in the case of a double exponential decay, phasors appear inside the universal circle on the line joining the phasors of the main components on the universal circle. The contribution/fraction of one single component to the lifetime is proportional to the distance of the phasor from the other pure component [41,44].

FLIM data have been analyzed using the SimFCS software (Laboratory for Fluorescence Dynamics, University of California, Irvine, CA, USA, available at www.lfd.uci.edu). For di-4-ANEPPDHQ and CF-TP10 measurements, the system was calibrated using the known properties of fluorescein at basic pH (a single exponential decay of 4.0 ns) [45]. Alexa_594_, which exhibits a single exponential decay of 3.9 ns [46], was used to calibrate the Nile Red lifetime measurements.

## 3. Results and Discussion

### 3.1. GPMVs Formation and Evaluation of GPMVs-TP10 Interaction

**Figure 1:**
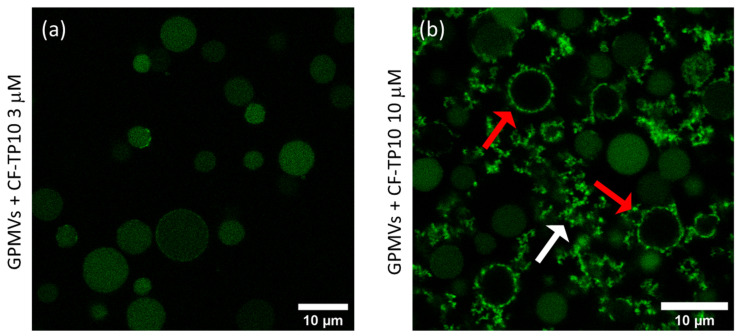
The 1024 × 1024 pixels representative LSCM measurements from GPMVs obtained from HepG2 cells after the addition of (**a**) 3 µM and (**b**) 10 µM CF-TP10 (λ_exc_ = 470 nm; fluorescence range 500–600 nm). Measurements were acquired after 20 h of incubation when the system was in stable conditions. Panel (**a**) reveals that the CF fluorescence is homogenously dispersed inside the GPMVs that retain their spherical shape. This suggests that the peptide translocates the membrane without affecting the morphology of the membrane. In panel (**b**), in addition to intact vesicles, the presence of morphologically modified vesicles is highlighted with red arrows, and the presence of amorphous aggregates, which could be the result of ruptured vesicles, is indicated with a white arrow. Experiments were performed in triplicates.

In Figure 1, the representative 1024 × 1024-pixel LSCM images of GPMVs after 20 h of incubation with (a) 3 μM and (b) 10 μM TP10 labeled with carboxyfluorescein (CF-TP10) are reported. The fluorescence signal of CF-TP10 is represented in green. Figure 1a shows the fluorescence signal colocalized with the GPMVs vesicles, which maintain a regular spherical shape and are quite homogenously colored in green. The GPMV shape is not changed with respect to that observed for the freshly prepared ones (see Figure S1 for comparison). The fluorescence signal is mainly localized within the vesicles indicating that TP10 crosses the membrane accessing the internal compartment. This is not surprising since TP10, being part of the CPPs family, is known in the literature to penetrate membranes alone or with molecular cargo [10,11,12,13,14]. Figure 1b reveals that at higher peptide concentrations (10 μM), although some vesicles retain their integrity, others (indicated by red arrows) present a clearly modified morphology. Furthermore, the white arrow highlights the presence of amorphous structures in the sample, characterized by higher intensity, which could represent lipid–peptide co-aggregates deriving from vesicle disruption. It is also interesting to observe in Figure 1b that the vesicles exhibiting a modified morphology (red arrows) do not show a fluorescence signal inside but present a higher fluorescence signal at the edges with respect to intact vesicles. This could result from an accumulation on/or insertion of the peptide on/in the bilayers coupled or not to efflux of the inner material, including the peptide. In line with previous results [13,30], it is possible to infer that this could be a consequence of the formation of pores in the membrane, which could also induce structural modifications including thinning and breakage. For example, experiments performed on DOPC:DOPG negatively charged giant unilamellar vesicles, using confocal fluorescence microscopy, showed the movement of the labeled peptide into the lumen, revealing the formation of pores and the occurrence of translocation phenomena [13]. All these processes require TP10–membrane interaction mediated by hydrophobic interactions and electrostatic forces due to the net positive charge of the peptide and the negatively charged lipids.

These measurements clearly demonstrated a different concentration-dependent mechanism of action of TP10. In particular, while at lower concentrations, TP10 crosses the membranes without modifying their morphology, at higher concentrations it alters the morphology of some vesicles, possibly inducing their disruption.

Next, to gain further information, double color experiments on the same system adding CF-TP10 to NR fluorescently labeled vesicles were performed.

**Figure 2:**
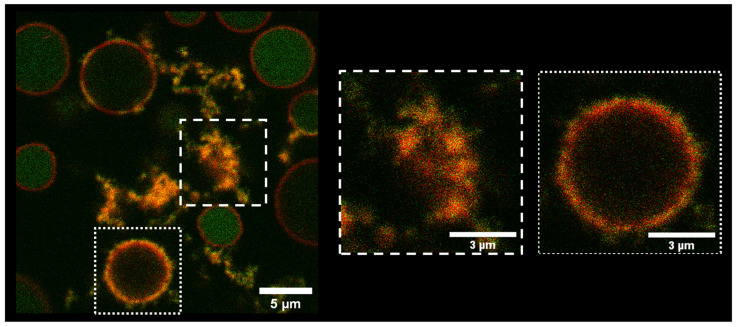
The 1024 × 1024 pixels representative fluorescence image shows GPMVs labeled with NR (red channel) after the addition of 10 μM CF-TP10 (green channel). The overlap of the two channels at the edges of the vesicles and in the aggregates in solution reveals protein–vesicles co-localization. This causes a clear modification in the membrane morphology (magnification highlighted with a dotted box) that can lead to the rupture of the membranes (magnification highlighted with a dotted box).

Figure 2 shows GPMVs labeled with NR (red channel) 20 h after the addition of 10 μM CF-TP10 (green channel). The fluorescence signals acquired in the two channels are clearly distinguishable in the intact vesicles with the green signal inside, delimited by the red one. On the contrary, a spatial overlap within the pixel resolution, between the green and red signal, is evident at the edges of the vesicles (highlighted with a dotted box) presenting a significantly modified morphology. This indicates that co-localization occurs (orange areas) between the lipid layer and TP10. As already observed in Figure 1b, the green fluorescent signal inside the vesicles with a modified membrane is lower with respect to the one on the lipid bilayer. This indicates the accumulation of peptides on the membrane.

Furthermore, these measurements also support the hypothesis that the amorphous structures observed in Figure 1b (here highlighted with a dashed box), are lipid–peptide co-aggregates. This proves that the vesicles which undergo morphology modification processes can also undergo rupture. To further analyze the interactions arising between TP10 and GPMVs, exploring both the fate of TP10 and the related consequences on the membrane in detail, FLIM measurements were performed. This method has the capability to combine the sensitivity of fluorescence spectroscopy with image information at pixel resolution, allowing us to map molecular interactions, gain molecular details on the environment around the fluorophore, and obtain information that is hardly accessible using intensity measurements alone [47].

### 3.2. FLIM Analysis on CF Fluorescence Lifetime to Evaluate the Fate of TP10

Measurements reported in this section gave us the chance to simultaneously follow the localization of TP10 and the mutual peptide–membrane interaction at the molecular level. Changes in the fluorescence lifetime of carboxyfluorescein take into account the changes in the environment around TP10 molecules. FLIM measurements were analyzed using the phasor approach (see Methods for details) [42,48]. This fitting-free analysis is much simpler than traditional approaches and provides a quick, detailed, and quantitative view of the data [42,43,48].

**Figure 3:**
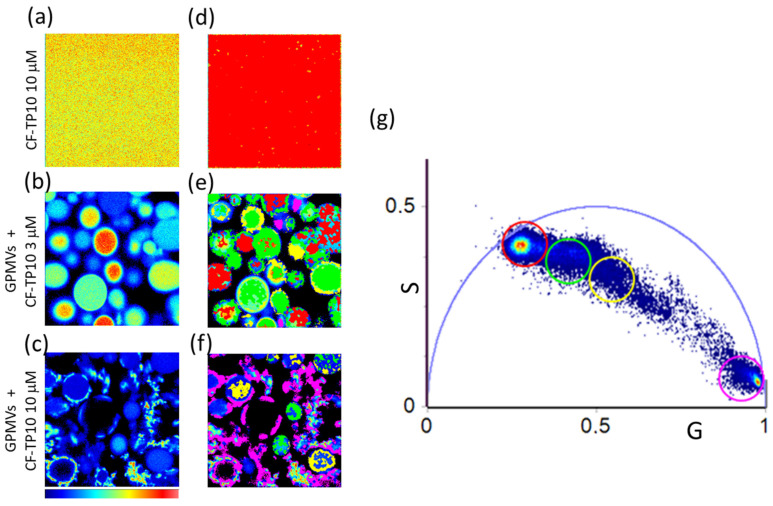
Phasor analysis of FLIM measurements on TP10 labeled with CF (λ_exc_ = 470 nm; fluorescence range of 500–650 nm). Intensity maps of (**a**) 10 µM CF-TP10 in GPMV buffer and (**b**,**c**) GPMVs 20 h after the addition of (**b**) 3 µM and (**c**) 10 µM CF-TP10 and corresponding lifetime maps (**d**–**f**) obtained by selecting lifetime distributions in the phasor plot (**g**). A cluster of pixels in the phasor plot was selected with red, green, yellow, and pink circles identifying corresponding pixels in the lifetime maps with the same color code.

In Figure 3, we report representative 256 × 256 pixels intensity images of (a) 10 µM CF-TP10 in solution and of GPMVs samples acquired 20 h after the addition of (b) 3 µM and (c) 10 µM CF-TP10. Panels (d–f) show the corresponding lifetime maps in which the pixels are colored with the same color as the cursors in the phasor plot in Figure 3g. The analysis of CF-TP10 fluorescence lifetime reveals a multifaceted scenario. The measured fluorescence lifetime in the presence of the membrane is characterized by shorter lifetimes with respect to the one measured for free peptide in the solution. Measurements in panels (a,d) reveal the uniform intensity and lifetime distribution, and all pixels in panel (d) are characterized by a lifetime distribution highlighted with a red cursor.

In the presence of vesicles, the fluorescence lifetime of CF-TP10 is critically modified. When GPMVs are in the presence of 3 µM TP10, a wide distribution in the lifetime is observed which can be analyzed using three colored cursors (red, green, and yellow) in the phasor plot. By observing the spatial distribution of the corresponding pixels in panel (e) it is possible to distinguish (i) regions where the fluorescence lifetime is the same as that measured in the absence of vesicles and (ii) regions, located in the lumen of the vesicles (green) and at the membrane (yellow), with lower lifetimes. In a previous study [7], on a simple two-component model membrane, we have ascribed a reduction in CF fluorescence lifetime to TP10 internalization in the bilayer and to the accumulation and aggregation of the peptide at the membrane surface. Here, larger changes are observed in parallel with a higher heterogeneity in the measured lifetime. This could be due to the higher complexity of the model system. TP10 could in fact interact with multiple constituents such as cytoplasm components, possibly resulting in short lifetimes highlighted in green and observed in the lumen, or proteins and lipids during its accumulation and/or insertion into the membranes (highlighted in yellow). The reduction in CF lifetime could be also ascribed to an accumulation/aggregation of the dye molecules, moving from the center to the edges of the vesicles, which induces CF quenching. These hypotheses are strengthened by observing results obtained for measurements on the sample incubated with 10 μM peptide, where a further lifetime distribution is found with a short lifetime, highlighted with the pink cursor. As evident in panels (c,f), the fluorescence signal, characterized by higher intensity and located at the membrane borders, also presents a shorter lifetime suggesting further compaction of the peptide environment and the occurrence of quenching phenomena. It is important to note that the observed changes begin to occur within one hour of incubation of GMPVs vesicles with TP10 (see Supplementary Information Figure S2); the peptide is internalized in the lumen with a consequent reduction in measured CF lifetime when interacting with biological components. This leads to progressive modifications in membrane shape towards the vesicle disruption observed after 20 h at a high peptide concentration. Using a map of the CF topology and lifetime, it is possible to locate peptide interaction sites, thus gaining information on the environment surrounding the dye.

To better understand the mechanisms that cause the membranes to lose integrity after the addition of 10 μM TP10, we analyzed the possible changes in the fluidity and the hydrophobicity of bilayers using an assessment of the fluorescence lifetime of di-4-ANEPPDHQ and NR dyes.

### 3.3. FLIM Analysis of Membrane Sensitive Dyes to Study TP10 Effects on the Physical Properties of the Membrane

#### 3.3.1. NR to Investigate Membrane Hydrophobicity

NR fluorescence properties change depending on the dielectric constant of its environment and have been previously studied to analyze how membrane order and fluidity are affected by lipid composition, chain saturation, and the presence of cholesterol [49,50,51,52]. It is well-known that changes toward a higher hydrophobic environment induce a blue shift in the emission spectrum of the dye as well as an increase in its lifetime [36].

**Figure 4:**
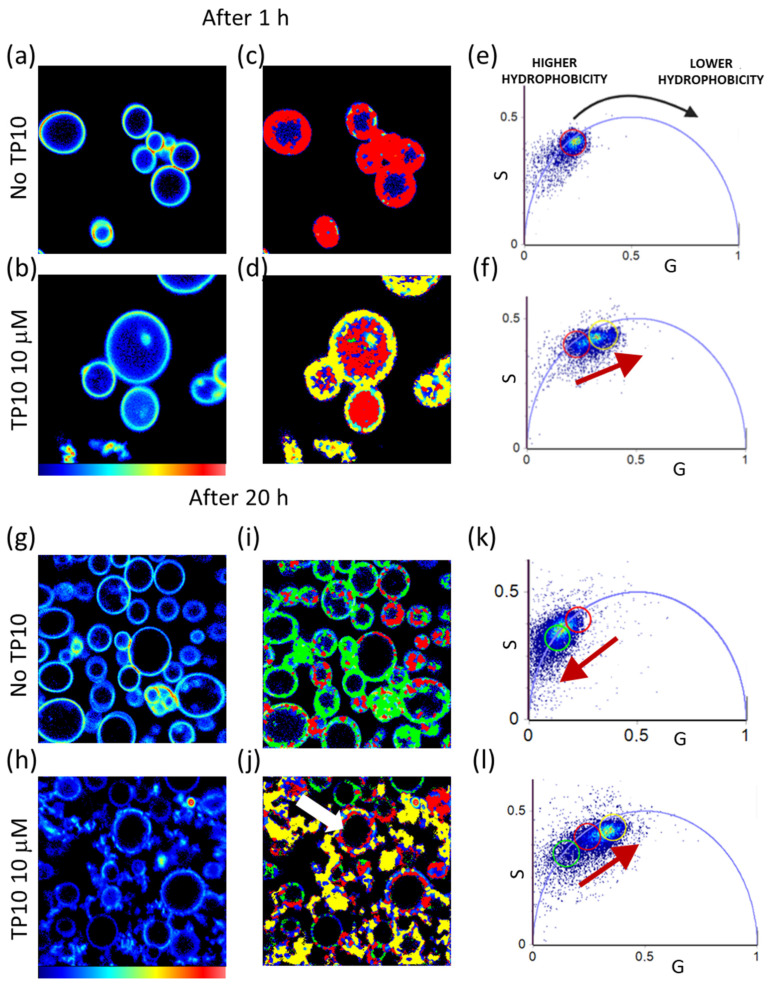
Phasor analysis of 256 × 256 pixels FLIM measurements from NR (λ_exc_ = 540 nm, range 580–700 nm) in GPMVs. Measurements acquired 1 h and 20 h after the addition of 10 μM TP10 are reported. (**a**) Fluorescence intensity map of a representative control measurement from NR-stained GPMVs in the absence of TP10. (**b**) Fluorescence intensity map for NR-stained GPMVs 1 h after the addition of 10 μM TP10. (**c**,**d**) Corresponding lifetime maps from the selection in the phasor plots in (**e**,**f**), respectively. (**g**) Fluorescence intensity map of a representative control measurement at the 20-h time point on NR-stained GPMVs in the absence of TP10. (**h**) Fluorescence intensity map for NR-stained GPMVs 20 h after the addition of 10 μM TP10. (**i**,**j**) Corresponding lifetime maps from the selection in the phasor plots in (**k**,**l**), respectively. Green, red, and yellow cursors are used to select different lifetime distributions and are kept in the same position.

In Figure 4a–b, we present 256 × 256 pixels NR fluorescence intensity maps in GPMVs in the absence of TP10 (a) and in the presence of 10 μM TP10 (b) 1 h after the peptide addition. In panels (c,d), we report the color maps in which each pixel of the images is colored according to the corresponding selection in the phasor plots (e,f). Intensity images carried out 20 h after the peptide addition are shown in Figure 4g–h, and the respective color maps are reported in (i–j) according to phasor plots in (k–l). In line with results in previous sections, measurements from NR-stained GPMVs, acquired after incubating them with 3 μM TP10, revealed no significant differences in the lifetime of the dye compared to the control (see Supplementary Information, Figure S3). Panels (a,g) show the control measurements from non-treated GPMVs in the same conditions.

As shown in the phasor plot in panel (e), a single exponential fluorescence lifetime distribution (3.7 ns) is observed for NR-stained GMPV in the absence of the peptide. By selecting the cloud using a red cursor, it is possible to highlight the membrane surface. In the presence of TP10, after 1 h of incubation, a broadening in the lifetime distribution is observed in the phasor plot (panel f) where the direction of the lifetime modification is indicated with a red arrow. It is possible to qualitatively describe it in terms of two different lifetime distributions highlighted using a red and a yellow cursor. The red cursor (at 3.7 ns) highlights the lifetime distribution characterized by a longer lifetime which superimposes to the one measured in the absence of the peptide (panel e). The yellow cursor is used to highlight a faster lifetime distribution. As evident in panel (d), pixels where the fluorescence lifetime is characterized by the lower lifetime are found at the membranes, while the ones corresponding to the longer lifetime are found within the lumen of the vesicles where a fluorescence signal is detected. This could indicate that NR, which is confined in the bilayer of untreated samples, is partially transferred in the internal cavity and interacts with cytoplasm constituents and with internalized TP10 molecules. The reduction in the fluorescence lifetime of NR at the edge of the vesicles, as a consequence of peptide interaction, could be caused by a decrease in the environmental hydrophobicity possibly due to an increase in the number of water molecules in the hydrophobic bilayer. This could be the result of an increase in membrane fluidity. The presence of intravesical material characterized by the same lifetime (red cursor) of the vesicles in the absence of peptide may also suggest the internalization of lipids in the lumen. Measurements performed on the control sample (shown in panels (g), (i), and (k)) after 20 h reveal that GPMVs are not stable structures but they spontaneously change over time. The lifetime distribution in the phasor plot (k) is broadened towards longer lifetimes; in the phasor plots, the variations are highlighted using the green cursor while the red cursor is maintained in the same position as in panel (e). The observed changes indicate that NR is predominantly exposed to a lipid environment characterized by a minor water content as the aging of the vesicles can lead to dehydration. Measurements performed 20 h after TP10 addition, shown in panels (h), (j), and (l), again reveal the increase in sample heterogeneity. Interestingly, the measured fluorescence lifetime change is in the opposite direction from that due to sample aging. In particular, there are few intact vesicles characterized by the same fluorescence lifetime (green and red cursors) measured for GPMVs in the absence of TP10, while most of the membranes, which are modified or destroyed, present a shorter lifetime (yellow cursor). Some vesicles, such as the one indicated with the white arrow in panel (l), appear still intact but with irregular profiles. In the phasor map, the corresponding pixels are colored red and yellow. TP10, by perturbing the membranes, could in fact increase the accessibility of water molecules at the membrane level, which could be also enhanced by a different osmotic pressure between the cytosolic environment and the external environment. The hydration process can lead the membranes to swell and burst, disintegrating them in fragments mostly highlighted and colored in yellow (lowest lifetime) in panel (j). An analogous representative measurement, acquired by zooming in on the area of interest on the single vesicle still intact but critically modified in shape, is reported in Supplementary Information Figure S4. This measurement was chosen in order to highlight membrane modifications before the rupture.

Summing up, measurements acquired from NR-stained GPMVs revealed that the vesicles are not stable over time and spontaneously undergo dehydration processes leading to an increase in the measured NR lifetime. TP10 addition induces critical modifications which also produce a reduction in NR lifetime. These changes were ascribed to increased water molecules accessibility within the membranes and thus the growth in the fluidity of the lipid bilayer.

#### 3.3.2. di-4-ANEPPDHQ to Investigate Membrane Fluidity

To confirm the previous hypothesis, membranes were marked with di-4-ANEPPDHQ and FLIM measurements were carried out. It is widely accepted that the emission spectra and fluorescence decay of di-4-ANEPPDHQ depend on the lipid phase organization [39,40,41]. In particular, when the lipids are in a disordered phase and the water molecules easily move into the bilayer, the lifetime of the dye is characterized by shorter values than when the dye is inserted in ordered/rigid lipid phase bilayers [39,41].

**Figure 5:**
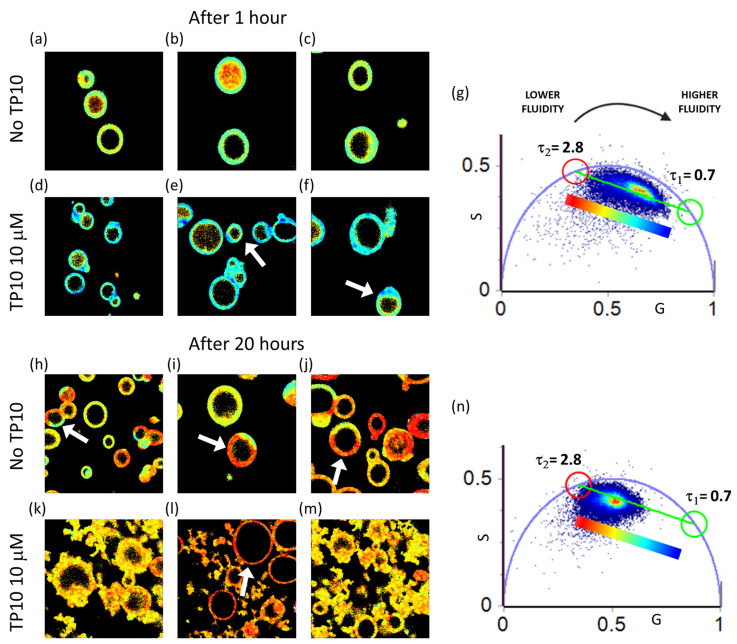
Phasor analysis of 256 × 256 pixels representative FLIM measurements from di-4-ANEPPDHQ (λ_exc_ = 470 nm, range 500–650)-stained GPMVs 1 h and 20 h after the addition of 10 μM TP10. (**a**–**c**) Lifetime maps of control measurements from di-4-ANEPPDHQ-stained GPMVs in the absence of TP10. (**d**–**f**) Lifetime maps of control measurements from di-4-ANEPPDHQ-stained GPMVs 1 h after the addition of 10 μM TP10. (**g**) Phasor plot from the analysis of all reported measurements performed after 1 h from the peptide addition. (**h**–**j**) Lifetime maps of control measurements at the 20-h time point from di-4-ANEPPDHQ-stained GPMVs in the absence of TP10. (**k**–**m**) Lifetime maps of di-4-ANEPPDHQ-stained GPMVs 20 h after the addition of 10 μM TP10. The white arrow in panel (**l**) is used to indicate an intact vesicle with unmodified morphology and low fluidity. (**n**) Phasor plot from the analysis of reported measurements after 20 h. The green and red cursors are used to identify the two principal lifetime components of a double exponential decay that describes the di-4-ANEPPDHQ lifetime in present conditions. The shorter lifetime is τ_1_ = 0.7 ns (green cursor), and the longer lifetime is τ_2_ = 2.8 ns (red cursor). This analysis is used to define a heuristic fluidity scale going from red (lower fluidity–longer lifetime) to blue (higher fluidity–shorter lifetime).

In Figure 5, the phasor analysis of FLIM measurements from di-4-ANEPPDHQ-stained GPMVs acquired 1 h and 20 h after the addition of 10 μM TP10 is reported. The 256 × 256 pixels images (phasor maps) of non-treated GPMVs stained with di-4-ANEPPDHQ are reported in panels (a–c) as a control, while the lifetime maps for GPMVs stained with di-4-ANEPPDHQ after 1 h incubation with 10 μM TP10 are reported in panels (d–f). The phasor plot for these data is reported in panel (g), and the color code of the lifetime maps is defined according to the analysis described below. Phasor maps carried out 20 h after the peptide addition are shown in Figure 5h–o and colored according to the phasor plots in (p). The corresponding intensity maps, both for the measurements carried out after 1 h and after 20 h from TP10 addition, are reported in Figure S5.

Looking at the phasor plot in Figure 5g, a broad lifetime distribution is observed within the universal circle. Interestingly, it appears to lie on a straight line, possibly representative of a double exponential decay. According to the literature [41], in cell membranes, this may stem from a complex environment around the dye in which two lipid phases co-exist at the sub-resolution space scale. The equilibrium between the ordered/rigid phase and the disordered/fluid phase is found to change as a result of vesicle aging and/or the action of the peptide in the bilayer. In particular, the lifetime distributions lie on a straight line that connects two single exponential lifetimes of τ_2_ = 2.8 ns and τ_1_ = 0.7 ns highlighted with red and green cursors, respectively. These two principal lifetime components were used for the decomposition of the decay using the following equation:

$$I\left(t\right)=F_{1}e^{-t/\tau_{1}}+F_{2}e^{-t/\tau_{2}}$$

The fraction (F) of each component is proportional to the distance between each point on the cloud in the phasor plot and the other single exponential phasor [44]. This analysis allows us to define a sort of “fluidity” scale which is used to map lifetime in Figure 5a–c,d–f. Images are colored in false colors according to the fractions F_1_ and F_2_ of the τ components. The scale goes from blue to red moving from the pure fast component (τ_1_ = 0.7 ns) to the pure slow component (τ_2_ = 2.8 ns) setting a fluidity scale going from more fluid to more rigid membrane configurations [39,53]. Panels (d–f) reveal that the addition of peptide induces a shift in the di-4-ANEPPDHQ lifetime towards shorter values, the fastest decay F_1_ is in fact dominant in GPMVs with added peptide (F_1_ ~ 0.79) if compared to the control (panels a–c) where F_1_ ~ 0.68. This means that the peptide induces an increase in the fluidity of the membrane, allowing more water molecules to move into the bilayer. This is in accordance with data in Figure 4, which showed a decrease in the hydrophobicity of the environment around the NR. Interestingly, in panels (e,f), it is possible to distinguish phase separation over a single vesicle (see, e.g., the ones indicated with white arrows). Pixels in which the blue component is dominant are characterized by shorter lifetimes, while the other pixels, mostly colored in green, present longer lifetime values. This means that the same structure can present different accessibility to water molecules. The phase separation in intact plasma membranes, isolated from live cells, has been already observed [35] and attributed to tighter packing of cholesterol with phospholipids containing long, saturated acyl chains (L_o_) than with phospholipids containing two or more double bonds in their acyl chains (L_d_) [54]. In this context, the presence of intrinsic proteins has to be taken in account as an important contributor to plasma membrane heterogeneity as specific protein/protein and protein/lipid interactions may stabilize membrane microdomains. However, the phase separation observed with di-4-ANEPPDHQ was not observed with NR, and this could suggest that peculiarities in the dyes provide different sensing opportunities. In particular, while the NR is a neutral probe, the di-4-ANEPPDHQ is negatively charged and thus may occupy different locations with respect to NR. This may affect its preferential location and then provide preferential information on the spatial reorganization of a sub-resolution charged environment which in this case may undergo phase separation.

Measurements performed after 20 h on the non-treated GPMVs (panels (h–l)) reveal what has already been demonstrated above using the measurements from NR-stained vesicles, i.e., that GPMVs undergo the dehydration processes over time.

In comparison to the phasor plot corresponding to measurements at the 1 h time point (panel g), the phasor plot reported in panel (n) shows a shift in the di-4-ANEPPDHQ lifetime distribution towards longer values. Interestingly, these representative measurements performed in the absence of peptide reveal also a clear phase separation in the membrane already observed in GPMVs with added TP10 (Figure 5e,f). The white arrows in Figure 5h–j highlight single vesicles presenting small regions where inhomogeneous lifetime is measured (red pixels, lower fluidity; green pixels, higher fluidity). In panels (k-m), measurements performed 20 h after TP10 addition show that the sample is characterized by a reduced fluidity, and in agreement with the experiments reported above, the coexistence of intact, modified, and disrupted vesicles is observed. Interestingly, the GMPVs which have retained their morphology (white arrow in panel (l)) are characterized by a quite uniform di-4-ANEPPDHQ fluorescence lifetime distribution corresponding to a more ordered phase (F_1_ ~ 0.61), and in contrast, the modified and disrupted (previously referred as protein-lipid co-aggregates) vesicles are characterized by a lower lifetime (F_1_ ~ 0.69).

In conclusion, the measurements from di-4-ANEPPDHQ-stained GPMVs confirmed that the vesicles undergo stiffening processes during aging and validated the previous hypothesis that TP10 induces an increase in membrane fluidity.

## 4. Conclusions

Membrane active peptides are attracting much interest in light of their ability to mediate cellular uptake and also to act as antibacterial [4,6,32] and antitumor agents themselves [10,25]. Here, we show the concentration-dependent effects of TP10, a widely studied peptide belonging to this family, on GPMVs derived from cancer liver cells. Fluorescence lifetime imaging was used to highlight changes that occurred at the molecular level both on peptides and on membranes. We have chosen two different peptide concentrations to highlight the partitioning between two critically different peptide behaviors: at lower concentrations, TP10 translocates into the lumen of vesicles without inducing any significant modification in the membrane, while at higher concentrations, it accumulates on the membrane inducing critical changes in the morphology of most of them, possibly favoring the internalization of membrane components and, in some cases, causing the vesicles to collapse. This different behavior depending on concentration could drive future studies aimed at evaluating the possible use of TP10 both in drug delivery and in anticancer therapies.

It is also interesting to note that at higher peptide concentrations, in analogous conditions, when TP10 is in presence of a simple negatively charged synthetic model membrane, neither the peptide internalization nor vesicle collapse was observed [7]. This highlights the critical role of membrane composition in modulating peptide–membrane interactions. Moreover, the data reported in this study revealed that morphology modification is accompanied by changes in fluidity. In particular, disruptive membrane–peptide interaction causes an increase in the fluidity of the membranes. The presence of cholesterol [55,56,57], charged lipid content [58], and proteins seem to regulate occurring phenomena together with the dominant role of water, hydration, or dehydration in different domains. Hydrophobicity and fluidity were monitored using FLIM measurements of NR and di-4-ANEPPDHQ, respectively. NR results suggest the possibility that the increase in water permeation could be also connected to the formation of pores as a consequence of TP10 insertion. These effects, in the cellular environment, may allow a high number of water molecules to permeate the bilayers leading to a decrease in the hydrophobicity and causing an alteration in water balance, which is essential for cell survival. We envisage the possible application of the presented methodologies and results in the future. For example, the comparison of results from GPMVs models originating from different cancer or non-cancer cell lines could give insights into key elements for the therapeutic application of this peptide. Moreover, due to the intrinsic ability of TP10 in delivering molecules within the cells, a new experimental platform could be designed to analyze the fate of different cargoes in a controlled cellular environment.

## Data Availability

All data contained within the manuscript and Supplementary Materials are available upon request to the corresponding author.

## Supplementary Materials

The following supporting information can be downloaded at https://www.mdpi.com/article/10.3390/biom13030492/s1, Figure S1: HepG2 cells before and after “vesiculation buffer” addition, Figure S2: FLIM measurements from CF-TP10 1 h after the addition to GPMVs, Figure S3: FLIM measurements from NR-stained GPMVs in the presence of lower TP10 concentration, Figure S4: FLIM measurements from NR-labeled GPMVs morphologically modified with the action of TP10, Figure S5: Intensity maps of FLIM measurements from di-4-ANEPPDHHQ-labeled GPMVs.
