# Supplementary material for: Transportan 10 Induces Perturbation and Pores Formation in Giant Plasma Membrane Vesicles Derived from Cancer Liver Cells
Source: Biomolecules. 2023 Mar 7;13(3):492. doi: 10.3390/biom13030492 (PMC10046094; doi:10.3390/biom13030492)
Supplement: Supplementary file 1 [file biomolecules-13-00492-s001.zip › biomolecules-2160284-supplementary.pdf]

biomolecules

MDPI

Article

Transportan 10 Induces Perturbation and Pores Formation in Giant Plasma Membrane Vesicles Derived from Cancer Liver Cells

Sara Anselmo [1], Giuseppe Sancataldo [1], Concetta Baiamonte [2,3], Giuseppe Pizzolanti [3,4] and Valeria Vetri [1,3,*]

1 Dipartimento di Fisica e Chimica-Emilio Segré, Università degli Studi di Palermo, 90128 Palermo, Italy
2 Dipartimento di Scienze e Tecnologie Biologiche Chimiche e Farmaceutiche, Università degli Studi di Palermo, 90128 Palermo, Italy
3 AteN Center-Advanced Technologies Network Center, Università degli Studi di Palermo, 90128 Palermo, Italy
4 Dipartimento di Promozione della Salute, Materno-Infantile, di Medicina Interna e Specialistica di Eccellenza "G. D'Alessandro", Università degli Studi di Palermo, 90127 Palermo, Italy
* Correspondence: valeria.vetri@unipa.it

Supporting Informations

HepG2 cells before and after "vesiculation buffer" addition

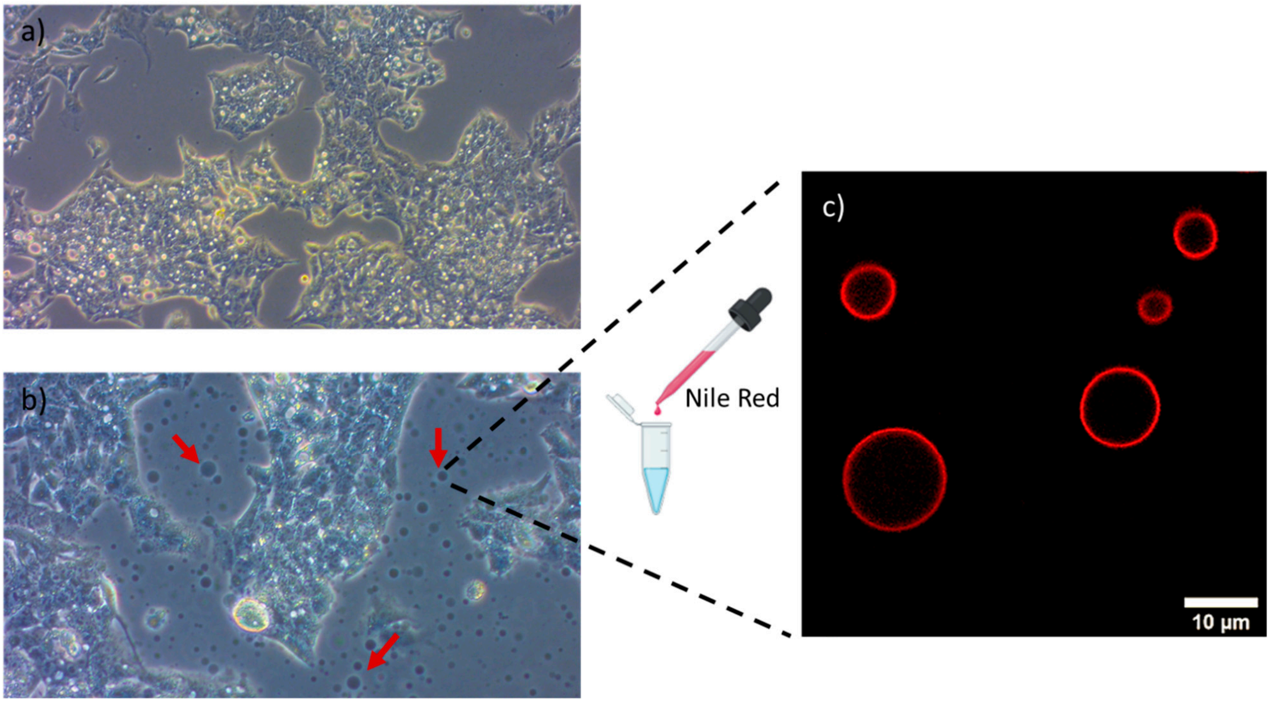

Figure S1. Images of HepG2 cells (a) before and (b) after "vesiculation buffer" addition. Vesiculation buffer induces the formation of spherical vesicles, highlighted by the red arrows. (c) 1024×1024 pixels representative LSCM measurements of the isolated GPMVs labelled with NR (λ_{exc}= 540 nm, range λ=580-700 nm).

FLIM measurements on CF-TP10 1 hour after the addition to GPMVs

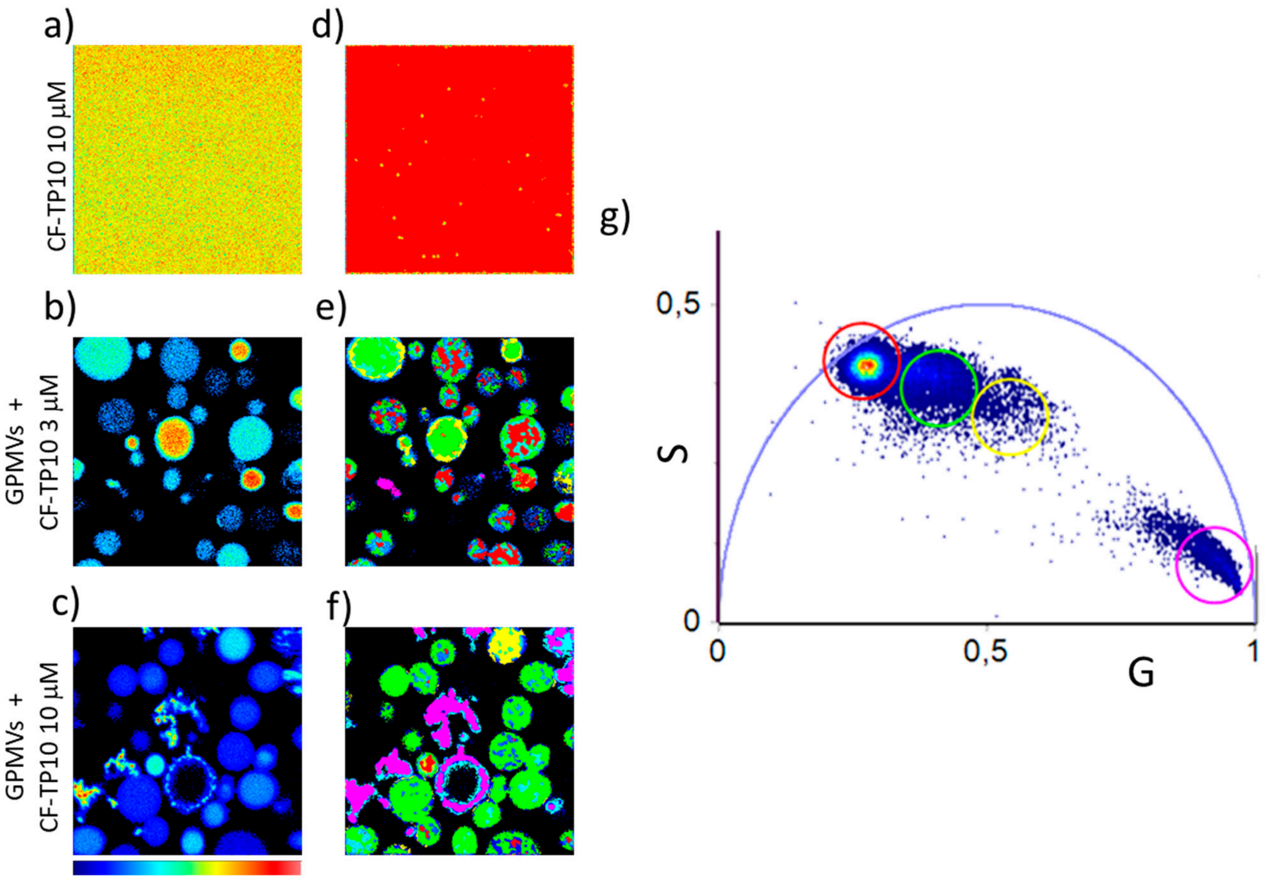

Figure S2. Phasor analysis of FLIM measurements on CF-labelled TP10. The signal was acquired under laser excitation at λ_{exc}=470 nm and collected in the range λ=500-650 nm. Intensity maps of (a) 10 µM CF-TP10 in GPMV buffer and (b and c) GPMVs 1 hours after the addition of (b) 3 µM and (c) 10 µM CF-TP10. (d-f) Lifetime maps corresponding to (a-c) measurements coloured according to the colour code obtained from the phasor plot (g). The choice of the size and the position of the circles is arbitrary.

FLIM measurements on NR stained GPMVs in presence of lower TP10 concentration

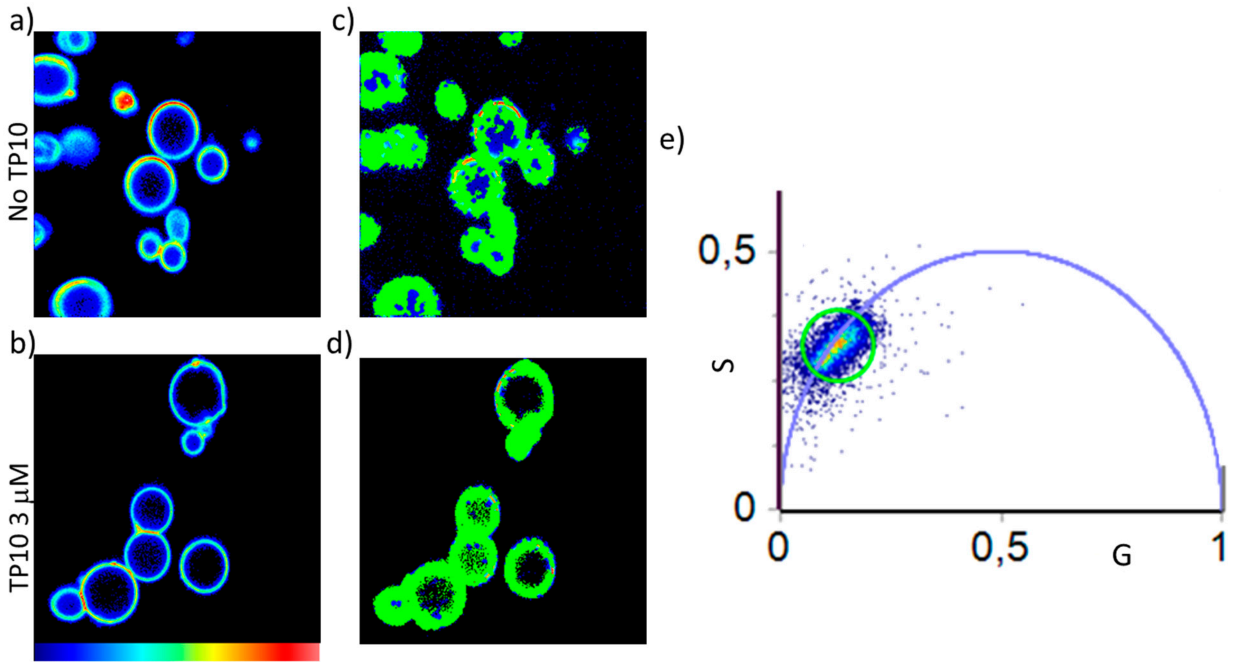

Figure S3. (a) and (b) 256 × 256 pixels fluorescence intensity images on NR (λ_{exc}= 540 nm, range λ=580-700 nm) in GPMVs, (b) 20 hours after TP10 3 µM addition. (a) the control measurement on NR labelled GPMVs, in absence of TP10, in the same time conditions. (c and d) The corresponding phasor colour maps in which each pixel is coloured according to the colour of the corresponding cursors in the phasor plot (e).

FLIM measurements on NR labelled GPMVs morphologically modified by the action of TP10

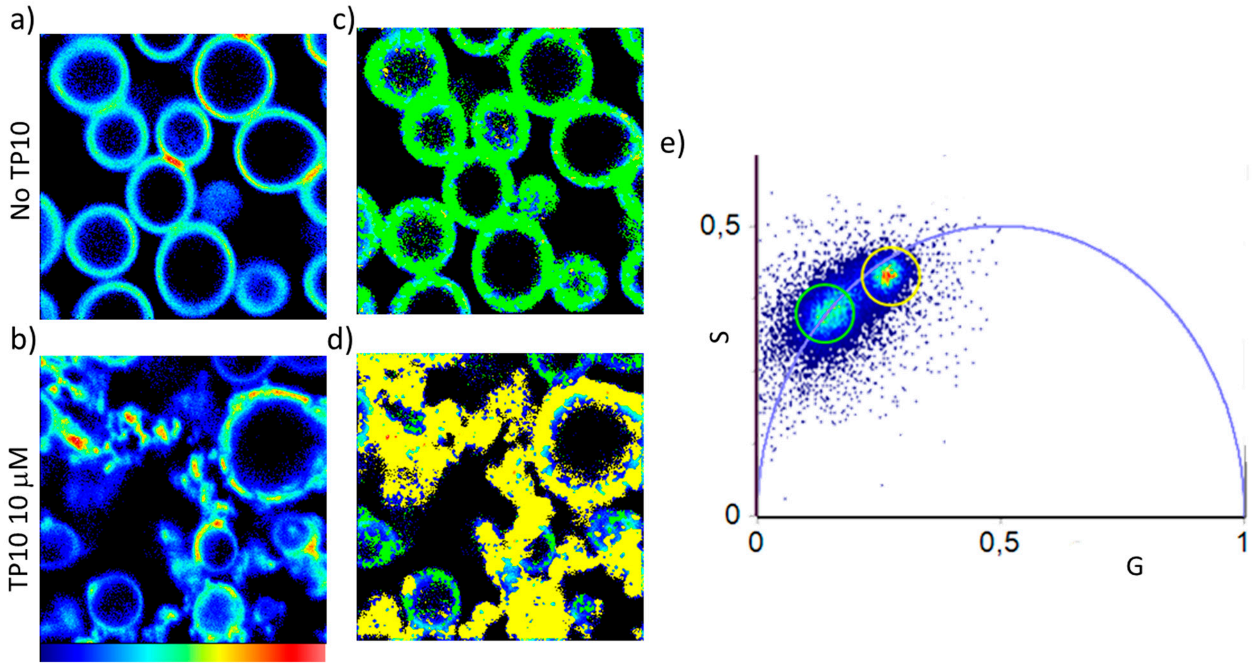

Figure S4. (a) and (b) 256 × 256 pixels fluorescence intensity images on NR (λ_{exc}= 540 nm, λ=range 580-700 nm) in GPMVs, (b) 20 hours after TP10 10 µM addition. (a) the control measurement on NR labelled GPMVs, in absence of TP10, in the same time conditions. (c and d) The corresponding phasor colour maps in which each pixel is coloured according to the colour of the corresponding cursors in the phasor plot (e).

Intensity maps of FLIM measurements on Di-4-ANEPPDHHQ labelled GPMVs

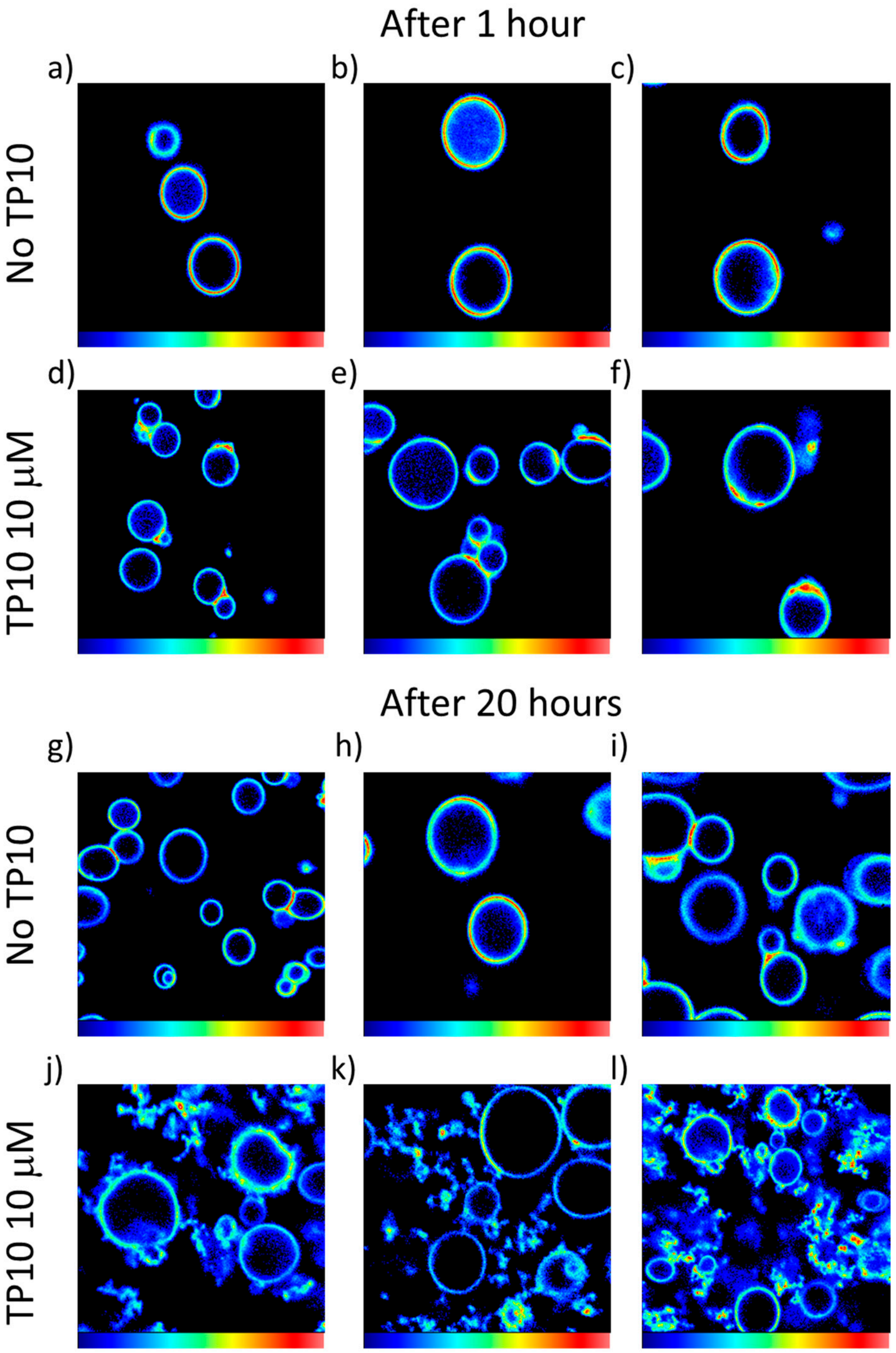

Figure S5. 256 × 256 pixels fluorescence representative intensity images on di-4-ANEPPDHQ (λ_{exc}= 470 nm, range λ=500-650) stained GPMVs (a-c) in the absence of TP10 and (d-f) after 1 hour from the addition of 10 µM TP10. 256 × 256 pixels intensity maps of representative (g-i) control measurements at 20 hours' time point on di-4-ANEPPDHQ stained GPMVs in the absence of TP10 and (j-l) on di-4-ANEPPDHQ stained GPMVs 20 hours after the addition of 10 µM TP10.
